# Eosinophilic esophagitis after congenital diaphragmatic hernia

**DOI:** 10.1186/s13052-016-0307-y

**Published:** 2016-11-08

**Authors:** Amelia Licari, Riccardo Castagnoli, Gian Luigi Marseglia

**Affiliations:** Department of Pediatrics, Foundation IRCCS Policlinico San Matteo, University of Pavia, Viale Golgi n. 19, 27100 Pavia, Italy

## Abstract

**Background:**

Eosinophilic esophagitis (EoE) is an increasingly diagnosed disease, especially in the western world. Although its pathogenesis remains poorly understood, there is strong evidence that the eosinophilic inflammation in EoE is primarily triggered by immune dysregulation secondary to allergic sensitization to dietary or aero-allergens. Recent studies have reported a higher prevalence of EoE in children with congenital gastrointestinal malformations, i.e. esophageal atresia and/or tracheoesophageal fistula.

**Case presentation:**

We present the case history of a 2-year-old boy who developed EoE in the aftermath of congenital diaphragmatic hernia (CDH) repair.

**Conclusions:**

To the best of our knowledge, the following case report describes for the first time the possible association between CDH and EoE. Given the increasing reported prevalence of EoE in children with congenital gastrointestinal malformations, EoE should be rule out also in CDH survivors.

## Background

Eosinophilic esophagitis (EoE) is an increasingly diagnosed disease in both pediatric and adult patients, especially in the western world [1]. EoE is currently defined as a chronic, immune/antigen-mediated esophageal disease characterized clinically by symptoms related to esophageal dysfunction and histologically by eosinophil-predominant inflammation [2]. Although the pathogenesis remains poorly understood, there is strong evidence that the eosinophilic inflammation in EoE is primarily triggered by immune dysregulation secondary to allergic sensitization to dietary or aero-allergens [3]. While adolescents and adults are more likely to suffer from dysphagia and episodes of food impaction, in children EoE may present with a wide variety of nonspecific, gastroesophageal reflux disease (GERD)-like symptoms, including feeding difficulty, nausea and vomiting, heartburn, and failure to thrive, and the index of suspicion for EoE should be raised in all patients in whom antireflux medication is unsuccessful in resolving symptoms [4].

Recent studies have reported a higher prevalence of EoE in children with congenital gastrointestinal malformations, i.e. esophageal atresia and/or tracheoesophageal fistula [5–7].

We present the case history of a patient who developed EoE in the aftermath of congenital diaphragmatic hernia (CDH) repair.

## Case presentation

A 2-year-old boy presented to our unit for recurrent vomiting associated with persistent cough.

His history was characterized by prenatal diagnosis of left CDH. He was born through an elective cesarean section at 37 weeks of gestation and immediately intubated and assisted with high-frequency ventilation. At 2 days of life, the surgical repair of CDH was performed. The procedure was executed without any complications and the patient recovered in neonatal intensive care unit for the first two months of life. However, at 1 month of life, for the detection of failure to thrive associated with vomiting and feeding difficulties, GERD, a well-recognized consequence of CDH, was diagnosed and an antireflux therapy (omeprazole, 10 mg daily) was started with initial improvement in symptoms.

When we evaluated the patient at 2 years of age, the persistence of esophageal symptoms unresponsive to prolonged antireflux therapy represented an indication for esophagogastroduodenoscopy. Endoscopy revealed only mucosal erythema in the lower half of the esophagus. Moreover, esophageal biopsy specimens taken from different parts of the esophagus showed a mean eosinophil count of 40 eosinophils per high-power field [HPF], allowing the final diagnosis of EoE. Complementary testing revealed an elevated serum immunoglobulin E level and positive skin-prick tests for egg, milk and fish. With the introduction of the targeted elimination diet and swallowed inhaled fluticasone (200 mcg daily), we observed symptoms resolution and both endoscopic and histologic remission.

## Discussion

To the best of our knowledge, this is the first literature report of EoE as a follow-up complication of CDH.

The outcome of newborns with CDH has improved rapidly with recent advances in perinatal intensive care, resulting in an increasing survival [8]; however, in follow-up studies of infants born with CDH, many complications including gastrointestinal problems have been described [8]. In particular, GERD is reported in up to 62 % of CDH survivors [9]. Many factors may contribute to the pathogenesis of GERD after CDH repair. The combination of increased abdominal-intrathoracic pressure gradient, resulting from positioning the hernial contents into the abdominal cavity during the repairing procedures, and the underdevelopment of the diaphragmatic crus may increase the strain on the crus facilitating the passage of gastric contents to the esophagus [10]. Moreover, abnormality of the esophageal dimensions might also contribute to development of gastroesophageal reflux [10]. Finally, there is evidence of abnormal enteric innervation in CDH and it is likely that esophago-gastric peristalsis is abnormal [10]. The clinical presentation of CDH-associated GERD may vary, including recurrent vomiting and regurgitation, recurrent episodes of bradycardia and respiratory arrest, failure to thrive and recurrent pneumonia [11]. In adult CDH survivors, incidence of esophagitis complicating GERD is 54 %, which is significantly higher than the expected 2 % of endoscopic assessed esophagitis in the general adult population [12]. Considering these data, a long-term follow-up for GERD, including endoscopic evaluation, is mandatory in CDH survivors.

The association between GERD and EoE has been long investigated. The exact interplay between these diseases remains unclear. However, possible explanations for the interaction between EoE and GERD include three major mechanisms. The first hypothesis speculates that both diseases coexist but unrelated: GERD has a high incidence in general population, affecting approximately 20 % of adult in western countries, so unrelated coexistence is very likely. Regarding the second pathogenetic mechanism, it has been demonstrated that EoE induces esophageal remodeling with tissue fibrosis that might affect lower esophageal sphincter, favoring GERD. Moreover, eosinophils produce several substances that alter esophageal motility, causing delayed esophageal clearance of refluxed material and increasing contact time with refluxed gastric juice, thus promoting the development of GERD. Finally, as third hypothesis, it is possible that GERD contributes to or causes EoE by inducing epithelial changes that predispose to EoE. It has been demonstrated that the normally impermeable esophageal mucosa, when exposed to acid, becomes permeable for peptides up to 20 kDa. Therefore, the deeper esophageal layers may become exposed to allergens that might cause EoE. In addition, GERD may contribute to esophageal eosinophilia by inducing the expression of eosinophil chemoattractants [13].

The diagnosis of EoE is defined by multiple criteria: symptoms related to esophageal dysfunction; histologic evidence of eosinophilia limited to the esophagus (and not in other parts of the gastrointestinal tract), where finding 15 esophageal intraepithelial eosinophils per HPF is accepted as the minimum threshold for diagnosis; the disease should remit with treatments of dietary exclusion, topical corticosteroids, or both; the esophageal eosinophilia should not be responsive to proton pump inhibitor (PPI) therapy alone [2].

Because the presenting symptoms are similar, many patients are initially thought to have GERD. The main histologic characteristic of EoE is the presence of eosinophils in the esophageal mucosa and submucosa, whereas eosinophils virtually are absent in the normal esophagus. Eosinophils may also be present in other conditions, especially reflux esophagitis. Although the esophageal eosinophil count in patients with GERD seldom surpasses 7 eosinophils/HPF, in EoE they reach far higher concentrations; in the proper clinical context, a peak count of more than 15 intraepithelial eosinophils/HPF is required for the diagnosis of EoE. For a definite differentiation from reflux esophagitis, it is suggested that symptoms and histology do not improve on adequate antireflux treatment, preferably with PPIs [2].

In our report, a 2-year-old boy developed EoE as a follow-up complication of CDH repair. The case meets the definition for EoE because the number of intraepithelial eosinophils was considerably higher than 15 and because antireflux treatment was unsuccessful. As previously theorized for other congenital gastrointestinal malformations, i.e. esophageal atresia and tracheoesophageal fistula, we speculate that, in CDH patients, GERD, a well-recognized consequence of CDH, could predispose to EoE by altering the esophageal mucosal permeability to allergens and by inducing the expression of eosinophil chemoattractants. Furthermore, after CDH repair, esophageal motility disturbances may prolong the exposure to potential allergens for the already-damaged mucosa, increasing the risk of local sensitization to potential antigens.

These observations suggest the possibility of considering EoE in CDH survivors with GERD-like symptoms and dysphagia refractory to antireflux medications.

The differential diagnosis between GERD and EoE is fundamental in order to set up a targeted therapy. In particular, EoE will not respond to antireflux therapy but only to dietary exclusion, topical corticosteroids, or both; finally anedoctal cases have reported the efficacy of omalizumab in the treatment of EoE [14].

## Conclusion

Given the increasing reported prevalence of EoE in children with congenital gastrointestinal malformations, EoE should be rule out also in CDH survivors before considering antireflux surgery for GERD refractory to drugs.

## Abbreviations

CDH: Congenital diaphragmatic hernia
EoE: Eosinophilic esophagitis
GERD: Gastroesophageal reflux disease
HPF: High-power field
PPI: Proton pump inhibitor.
